# Tissue response and expression of interleukins (IL)-1ß, IL-6, IL-10 after pulp capping with bioglasses in mice

**DOI:** 10.1590/1807-3107bor-2024.vol38.0096

**Published:** 2024-12-09

**Authors:** Hebertt Gonzaga dos Santos Chaves, Barbara Figueiredo, Caroline Andrade Maia, Alexandre Henrique dos Reis-Prado, Maísa Mota Antunes, Ricardo Alves de Mesquita, Warley Luciano Fonseca Tavares, Gustavo Batista Menezes, Ivana Márcia Alves Diniz, Murilo Camuri Crovace, Gleide Fernandes de Avelar, Francine Benetti

**Affiliations:** a Universidade Federal de Minas Gerias – UFMG, School of Dentistry, Restorative Dentistry, Belo Horizonte, MG, Brazil.; b Universidade Federal de Minas Gerias – UFMG, Biological Science Institute, Department of Morphology, Belo Horizonte, MG, Brazil.; c Universidade Federal de Minas Gerias – UFMG, School of Dentistry, Department of Oral Surgery and Pathology, Belo Horizonte, MG, Brazil; d Universidade Federal de São Carlos – UFSCar, Bioactive Materials Laboratory, São Carlos, SP, Brazil.

**Keywords:** Models, Animal, Dental Pulp Capping, Pulpotomy

## Abstract

This study aimed to evaluate the pulp response to F18 and cobalt-doped F18 bioglass (F18Co) in comparison with calcium hydroxide (CH) after pulp capping. The maxillary first molars of 48 rats were divided into F18, F18Co, CH, and control (no intervention) groups. The pulp was exposed, the materials were placed, and the teeth were capped. After 7 and 15 days, the animals were euthanized for pulp evaluation and interleukin (IL) expression determination. Statistical analysis was carried out using the SigmaPlot® program (Systat Software Inc., for Windows, version 12.0). The data obtained in the analyses were subjected to the non-parametric Kruskal-Wallis test, followed by Dunn's test. For all tests, statistical significance was set at p < 0.05. The CH group exhibited mild to moderate inflammation, whereas the bioglass groups displayed moderate to severe inflammation, indicating a notable difference between the control and bioglass groups. At 7 days, both the CH and most of the bioglass specimens showed moderate disorganization. On day 15, CH displayed mildto-moderate disorganization, whereas F18 and F18Co exhibited significantly more moderate-to-severe disorganization. There were no significant differences in IL-6 and IL-10 expressions between groups at 7 days, but a noteworthy increase in IL-1β was observed in both CH and F18. After 15 days, there was a greater expression of IL-6 and IL-1β in the bioglass groups. No significant IL-10 expression was observed. Bioglass performed less effectively than CH when in direct contact with the pulp tissue.

## Introduction

As a minimally invasive approach, vital pulp therapy (VPT) includes stepwise excavation of decayed tissues, direct/indirect pulp capping, partial/full pulpotomy, and partial pulpectomy.^1^ According to American Academy of Pediatric Dentistry, VPT is defined as a treatment that aims to preserve and maintain pulp tissue that has been compromised but not destroyed by caries, trauma, or restorative procedures in a healthy state. This is particularly important in young adult teeth with incomplete apical root development.^2^

Dental capping agents are materials used as a protective layer for the exposed pulp of the tooth to allow the tissue to recover and maintain its normal function and vitality.^3^ Ideally, these materials should be bioactive to the tissues, stimulating the migration, proliferation, and differentiation of the cells.^4^ Calcium hydroxide (CH) has been used as a direct pulp capping material for several decades.^5^ This material has a pH of approximately 12, which provides excellent antibacterial properties.^2,5^ Additionally, this pH induces the formation of a thin layer of tissue necrosis followed by tissue mineralization.^2^

Bioactive glasses, one of the bioceramics composed by silica (SiO_2_), sodium oxide (Na_2_O), calcium oxide (CaO), and phosphorus pentoxide (P_2_O_5_),^6^ show an ability to bond with bone through the formation of a hydroxyapatite layer on their surface.^2^ Thus, the use of bioactive glass was proposed to improve osseointegration, and new areas of research involving this material have emerged.^7^

A new highly reactive bioglass formulation that does not crystallize during processing has been developed and named F18.^8-11^ This material was developed with the objective of regenerating soft tissues, stimulating the proliferation of fibroblasts, and being potentially biodegradable.^8^ Thus, its potential to act in the repair of pulp tissue stands out. In a previous study, we observed that pastes of F18 showed biocompatibility, osteogenesis induction, and antimicrobial activity comparable to CH paste.^12^

However, there are still no studies that have evaluated the pulp tissue after direct capping with this bioglass. It is known that the addition of cobalt ions to the vitreous bioparticles induces tissue hypoxia and stimulates angiogenesis.^13^ Therefore, F18 bioglass was doped with cobalt ions (F18Co). This study evaluated the response of the pulp tissue to F18 and F18Co bioglasses compared to CH after direct pulp capping in mice. The null hypothesis that there would be no difference between the pulp response to different materials was adopted.

## Methods

Forty-eight male mice (Balb/C; 20 g and 8 weeks old) were used. The sample size calculation was based on a previous study.^14^ Considering an alpha error of 0.05% and sample power of 90%, a total of 6 animals per group were considered necessary for this study, as observed in other previous studies.^15,16^

The animals were kept in an environment with controlled temperature (22ºC– 24ºC) and light cycle (12 hours light/dark), with food and water *ad libitum*. The study was approved by the local Animal Research Ethics Committee (CEUA 08/2020) and conducted in accordance with the Guide for the Care and Use of Laboratory Animals of the National Institutes of Health (Bethesda, USA).

### Pulp exposure and direct pulp capping

The animals were anesthetized with 2% xylazine (10 mg/kg) and 10% ketamine (80 mg/kg) intraperitoneally. The two upper first molars (right and left) of each animal were randomly divided into 4 groups (n = 6): F18, F18Co, CH, and control (no intervention). Prior to coronary access, the first molars of the experimental groups were disinfected with 0.12% chlorhexidine digluconate.

Using a microscope (Alliance, São Paulo, Brazil), access to the pulp chamber was performed with an LN drill (Dentsply Maillefer, São José, Brazil) under constant irrigation with 0.9% saline solution. Then, using a K#15 endodontic file (VDW GmbH, Munich, Germany), careful drilling of the dentine was performed until the coronal pulp was exposed.^17^ Irrigation was performed with sterile saline solution. Bleeding was controlled with saline irrigation using sterile cotton balls. The pulp tissue then received one of the material pastes (F18, F18Co, or CH; prepared by spatulating each powder with distilled water, in a ratio 2:1 by weight)^12^ and was sealed with glass ionomer (Riva Light Cure – SDI, Indústria-Comercio LTDA, São Paulo, Brazil). The control group received no intervention. For postoperative analgesia, the rats received a single subcutaneous application of 150 mg/Kg of dipyrone.

### Sample preparation and histological analysis

After 7 and 15 days,^18^ the animals were euthanized by anesthetic solution overdose. The right and left maxillae were separated, and half of the hemimaxillae of each group, after removing the surrounding tissue, were stored and frozen in a freezer at −70ºC (Trammit Medical, Belo Horizonte, Brazil) for further analysis of cytokine expression. The other half was dissected, fixed in a solution of 4% buffered formaldehyde for 24 hours, and then decalcified in 10% acid ethylenediaminetetraacetic (EDTA) solution for approximately 30 days.

For histological processing, the jaws were dehydrated and embedded in paraffin. Serial histological sections (5-µm) were cut in the mesiosagittal plane and selected from the point where the mesial root of the first molar was seen in all its longitudinal extension. The histological sections were then stained with Hematoxylin-Eosin (HE). The analyses were performed under light microscopy (400× magnification; DM4000 B; Leica Microsystems, Wetzlar, Germany) by a single calibrated and blinded operator to the groups. To apply the scores and standardize the area to be evaluated, the region of pulp tissue immediately closest to the region where the material came in contact (middle region of the coronal pulp, in the mesiodistal direction) was analyzed. The pulp tissue was analyzed and scored according to histopathological parameters involving inflammatory infiltrate (0, inflammatory cells absent or negligible in number; 1, mild inflammatory infiltrate [<25 cells per field]; 2, moderate inflammatory infiltrate [between 25 and 125 cells per field]; 3, severe inflammatory infiltrate [>125 cells per field]; and 4, tissue necrosis) and pulp tissue disorganization (0, normal pulp tissue; 1, disorganization of the odontoblastic layer, but normal coronary pulp; 2, disorganization of the odontoblastic layer and part of the coronary pulp; 3, total disorganization of pulp morphology; 4, tissue necrosis).^19^

### Gene expression by real-time PCR

Total RNA from each sample was obtained from the first molar in reduced hemimaxillae using the ReliaPrep RNA Tissue Miniprep System (Promega, Durham, NC, USA) following the manufacturer's recommendations, and the RNA was quantified using a on NanoDrop spectrophotometer. Reverse transcription of RNA was performed using the SuperScript^®^ III Reverse Transcriptase kit (Life Technologies^®^, Carlsbad, USA) as per the manufacturer's protocol. The resulting cDNAs were amplified by PCR with SYBR^®^ Green qPCR SuperMix solution (Invitrogen^TM^, Massachusetts, USA). Fold increases for each sample were calculated using the ^ΔΔ^Ct method, with glyceraldehyde-3-phosphate dehydrogenase (GAPDH) as the endogenous gene for normalization. The primers used were:

- GAPDH: *forward* 5′CGACCACTTTGTCAAGCTCA3′, *reverse* 5′ GAGGGTCTCTCTCTTCCTCT 3′;
- IL-1β: *forward* 5′ GCCTCGTGCTGTCGGACCCATAT 3′, *reverse* 5′ TCCTTTGAGGCCCAAGGCCACA’;
- IL-6: *forward* 5′ CAACGATGATGCACTTGCAG3′, *reverse* 5′ GAAATTGGGGTAGGAAGGAC 3’;
- IL-10: *forward* 5′ ACTGGCTGGAGTGAAGACCA 3′, *reverse* 5′ AAGGCTTGGCAACCCAAGTAA 3’.

### Statistical analysis

The data were analyzed using the Kruskal-Wallis test followed by Dunn's post hoc test (p < 0.05).

## Results

### Histological analysis

**Figure 1:**
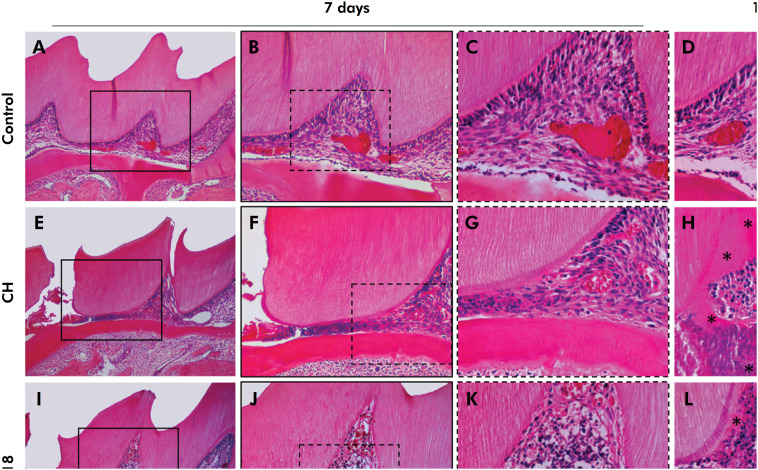
Representative images of pulp tissue reactions. (a-d) Control group with normal pulp tissue, absence of inflammatory cells and well-defined tissue organization. (e-h) CH group at 7 and 15 days; (e) it is possible to observe the region where the pulp exposure was performed and the insertion of the repair material, and (f, g) the presence of inflammatory cells and disorganization of the odontoblastic layer, in addition to the presence of blood vessels and center of pulp tissue with characteristics close to normal; (h) at 15 days, it is possible to observe the presence of tertiary dentine, in addition to odontoblast-like cells around this dentine. (i-l) F18 and (m-p) F18Co groups at 7 and 15 days, with a greater change in the pulp tissue visibly observed when compared to the CH group; (j, k, n, o) there is still a greater increase in the number of inflammatory cells and greater disorganization of the pulp tissue, in addition to the presence of blood vessels and the presence of some odontoblasts around the dentine; (l, p) presence of cells is observed, however, not so organized when observed in the CH group; the presence of blood vessels and less tertiary dentine formation are also noted. The symbol * indicates tertiary dentine. [H.E. staining; 50×, 200×, 400×]

Representative images of the histological analysis can be seen in Figure 1 *,* and the results are shown in Table 1. At 7 days, the CH group exhibited moderate inflammation in the pulp tissue, with the presence of polymorphonuclear cells and leukocytes, while the F18 and F18Co groups had moderate to severe inflammation. Regarding tissue disorganization, moderate disorganization was observed for the CH group; the F18 and F18Co groups showed disorganization of the odontoblastic layer and part of the coronal pulp, leading to total disorganization of the pulp morphology. A significant difference was observed only between the control group and the F18 and F18Co groups (p < 0.05).

**Table:** Histologic evaluation for each parameter in experimental groups at 7 and 15 days.

Scores for each parameter	7 days (n = 6)				15 days (n = 6)			
	Control	CH	F18	F18Co	Control	CH	F18	F18Co
Inflammatory infiltrate										
0	6/6	0/6	0/6	0/6	6/6	0/6	0/6	0/6
1	0/6	2/6	1/6	0/6	0/6	3/6	0/6	0/6
2	0/6	4/6	3/6	3/6	0/6	3/6	3/6	3/6
3	0/6	0/6	2/6	3/6	0/6	0/6	3/6	3/6
4	0/6	0/6	0/6	1/6	0/6	0/6	0/6	0/6
Median	0^a^	2^ab^	2^b^	2^b^	0^a^	1,5^ab^	2,5^b^	2,5^b^
p-value**	< 0.001				< 0.001			
Pulp tissue disorganization										
0	6/6	0/6	0/0	0/0	6/6	0/6	0/6	0/6
1	0/6	0/6	0/0	0/0	0/6	3/6	0/6	0/6
2	0/6	6/6	4/6	3/6	0/6	3/6	3/6	3/6
3	0/6	0/6	2/6	2/6	0/6	0/6	3/6	3/6
4	0/6	0/6	0/6	1/6	0/6	0/6	0/6	0/6
Median*	0^a^	2^ab^	2^b^	2,5^b^	0^a^	1,5^ab^	2,5^b^	2,5^b^
p-value **	< 0.001				< 0.001			

CH: calcium hydroxide; F18Co: F18 bioglass dopad with cobalt.

* Different letters in the same line indicate a statistically significant difference between groups in each period (p < 0.05);

** Kruskal–Wallis test followed by Dunn's test (significance was set at p < 0.05).

At 15 days, the CH group exhibited mild to moderate inflammation, while the F18 and F18Co groups had moderate to severe inflammation. Mild to moderate pulp tissue disorganization was observed for the CH group, whereas the F18 and F18Co groups exhibited moderate to severe disorganization. Again, a significant difference was observed between the control group and the F18 and F18Co groups (p < 0.05). Tertiary dentine was observed only at 15 days.

### Cytokines expression

**Figure 2:**
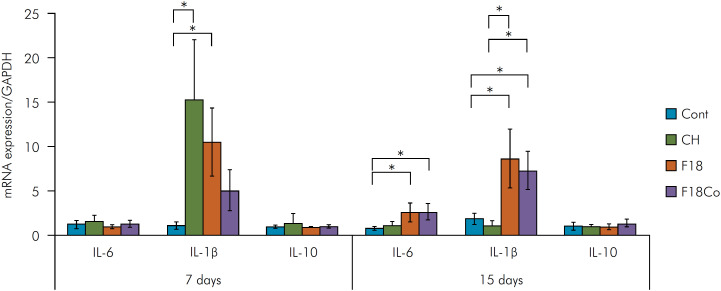
Representative graph of cytokine expression. At 7 days, there was no significant difference between groups regarding IL-6 and IL-10 expressions. Regarding the expression of IL-1β, it is possible to observe a significant increase in the CH and F18 groups. At 15 days, there was greater expression of IL-6 in F18 and F18Co groups compared to control. A significant increase in IL-1β expression was observed in this period in the F18 and F18Co groups compared to the CH and control groups. There was no significant expression of IL-10 in the different groups. The symbol * indicates significant difference between groups (p < 0.05).

The results of the cytokine expression analysis can be seen in Figure 2. At 7 days, there was no significant difference in IL-6 and IL-10 expression among the groups (p > 0.05), while the expression of IL-1β was significantly higher in the CH and F18 groups compared to the control (p < 0.05); the CH group showed the highest IL-1β expression, followed by the F18 and F18Co groups.

At 15 days, a significantly higher IL-6 expression was observed in the F18 and F18Co groups compared to the control (p < 0.05). Regarding IL-1β expression, there was a significant reduction in the CH group compared to the F18 and F18Co groups, which also showed higher IL-1β expression compared to the control (p < 0.05). No significant difference in IL-10 expression was observed (p > 0.05).

## Discussion

This study evaluated the pulp tissue response to F18 and F18Co bioglass pastes after direct pulp capping in mice molars, compared to CH paste. Overall, less inflammation and disorganization were observed in the CH group compared to the bioglasses, although no significant difference was found between the material groups. However, a difference was observed between the control group and the F18 and F18Co groups. The expression of IL-1β was higher in the CH and F18 groups at initial periods, but the IL-6 and IL-1β expressions were significantly higher at 15 days only in the bioglasses groups. There was no significant expression of IL-10 at any period of analysis. Thus, the null hypothesis was rejected regarding histological data and IL-6 and IL-1β expression, but accepted regarding IL-10 data.

It is known that mineral trioxide aggregate (MTA) is considered the gold standard material for pulpotomy and direct pulp capping.^20^ However, MTA does not allow the region where tissue was lost to be replaced by new tissue. Thus, CH was the most suitable material for comparison with bioglasses. Studies have shown that F18 is capable of stimulating fibroblast proliferation and is potentially biodegradable.^8^ De Araujo-Lopes *et al*.^12^ evaluated F18 as a paste in odontology, demonstrating that F18 has the ability to induce greater expression of osteopontin than CH.

F18 is highly bioactive and can form a layer of hydroxycarbonate apatite when in contact with tissue, which means it has a higher mineralization capacity compared to CH. Previous data revealed that F18 was efficient against gram-positive and gram-negative strains (*S. aureus, S. epidermidis, E. coli and P. aeruginosa*).^10^ Furthermore, De Araújo-Lopes *et al*.^12^ also showed activity against *E. faecalis*. Thus, the potential of this biomaterial to act in the repair of pulp tissue, as desired in pulpotomy or direct pulp capping procedures, is highlighted.

When CH is placed on the pulp tissue, a repair process occurs, characterized by the formation of a dentinal bridge, protecting the exposed tissue.^21^ Komabayashi *et al*.^22^ stated that the effect of CH is the result of the chemical injury caused by the release of hydroxyl ions when in direct contact with the pulp tissue, where slight inflammation caused by superficial necrosis is observed at different times of analysis. Previous studies have demonstrated more intense inflammation associated with CH when compared to the gold standard material.^2,3^

This is the first recent study that compared CH with bioglass in contact with the pulp tissue. The histological data showed less pulp inflammation and tissue disorganization with CH. In relation to bioglasses, the pulp tissue showed disorganization even at 15 days, with more pronounced inflammation, regardless of the presence of cobalt ions. Although all materials allowed the formation of tertiary dentine at 15 days, this was mainly observed in the CH group. However, this may indicate that a later analysis period could show a greater amount of dentine bridging with the bioglass, which still needs to be evaluated.

Furthermore, the differences found in the histological results between the CH group and the bioglass groups may be due to the particle size of the materials. While CH has particles around 2.5 µm, bioglasses were prepared with particles below 10 µm. Thus, larger particles of bioglasses would be in contact with the tissue compared to CH. Smaller particles of bioglass powders should be obtained to observe if there are better results.

Another fact that may explain the differences in the results is the reabsorption period of the materials. Bioglasses are reabsorbed over a longer period, while CH can be easily carried away by tissue fluids. The use of CH showed gradual dissolution, which can lead to the formation of a dead space and microleakage.^22^ A previous study that evaluated F18 fibers in the subcutaneous tissue of rats observed that at 15 days, F18 had been partially reabsorbed. However, reabsorption was greater at 30 and 60 days, while particles of the biomaterial were still found during these periods.^8^ In addition, cobalt ions have the ability to induce the formation of new vessels through the angiogenesis process,^23^ but this analysis was not possible due to tissue disorganization.

Cytokine analysis may be a good prognostic marker in teeth after pulp capping.^24^ This study also evaluated pro-inflammatory (IL-6 and IL-1β) and anti-inflammatory (IL-10) interleukins in pulp tissue after contact with the materials. Among the interleukins, IL-6 is an important regulator of inflammation, and its levels are increased in pulp inflammatory processes.^24^ It is a multifunctional inflammatory cytokine synthesized in response to trauma or infection, which can activate specific cells that play an important role in the inflammatory reaction and bone resorption.^26^ A previous study found elevated IL-6 immunolabeling in early periods (24 to 72 hours)^25^, which did not occur in our study. The increased IL-6 expression in the present study at 15 days may indicate that inflammation is still present during this period, with no reduction. This increase in IL-6 was not observed in the CH group. This can also be explained by the fact that the model used in this study did not have a previous induction model of inflammation before the application of the capping material.

Regarding IL-1β, this cytokine increased in a late period (at 60 days) in a previous study with all capping materials evaluated.^27^ These data are not consistent with the present study, in which the expression of this cytokine was increased in the F18 and CH groups in the initial period (7 days), but was significantly reduced later for CH. At 15 days, it was also increased for the F18Co group. This may indicate a reduction of inflammation over time after capping with CH, and highlights once again the need to assess more deeply and cautiously the pulp response to bioglasses.

Since IL-10 is an anti-inflammatory cytokine, its increase could be expected in the present study when performing pulp capping with bioactive materials. However, a previous study has shown that IL-10 was found mainly at the beginning of pulp inflammation when it was considered reversible.^28^ In this study, IL-10 was not significantly found in any period of analysis, which disagrees with another study, where there was an increase in IL-10 after contact of bioceramic materials with pulp cells.^29^ However, the difference in the experimental models used should be noted.

A limitation of this study was the fact that pulp capping was performed in an animal model of healthy pulp, without inflammation. Clinically, pulpotomy and pulp capping are indicated for teeth with inflamed pulp due to carious lesions in the young population. However, traumatic dental injuries involving the pulp are quite common and often lead to pulp exposure,^30^ sometimes treated without contamination. It is important for studies like this to perform an inflammation model before pulpotomy. Another limitation was the limited pulp content that is in contact with the tested material. The animal model is superior when compared to cell culture; however, it cannot be extrapolated to clinical practice. Therefore, it is necessary to consider the possibility that a given murine model response may not occur the same way in humans, necessitating studies with larger animal models.

## Conclusion

It is concluded that F18 and F18Co bioglass pastes may have lower performance than CH paste when used in direct contact with pulp tissue.
